# Effects of Information Visualization on Older Adults’ Decision-Making Performance in a Medicare Plan Selection Task: A Comparative Usability Study

**DOI:** 10.2196/humanfactors.5106

**Published:** 2016-06-01

**Authors:** Margaux M Price, Jessica J Crumley-Branyon, William R Leidheiser, Richard Pak

**Affiliations:** ^1^ John Deere Urbandale, IA United States; ^2^ Clemson University Department of Psychology Clemson, SC United States

**Keywords:** Information visualization, aging, health-related websites, working memory

## Abstract

**Background:**

Technology gains have improved tools for evaluating complex tasks by providing environmental supports (ES) that increase ease of use and improve performance outcomes through the use of information visualizations (info-vis). Complex info-vis emphasize the need to understand individual differences in abilities of target users, the key cognitive abilities needed to execute a decision task, and the graphical elements that can serve as the most effective ES. Older adults may be one such target user group that would benefit from increased ES to mitigate specific declines in cognitive abilities. For example, choosing a prescription drug plan is a necessary and complex task that can impact quality of life if the wrong choice is made. The decision to enroll in one plan over another can involve comparing over 15 plans across many categories. Within this context, the large amount of complex information and reduced working memory capacity puts older adults’ decision making at a disadvantage. An intentionally designed ES, such as an info-vis that reduces working memory demand, may assist older adults in making the most effective decision among many options.

**Objective:**

The objective of this study is to examine whether the use of an info-vis can lower working memory demands and positively affect complex decision-making performance of older adults in the context of choosing a Medicare prescription drug plan.

**Methods:**

Participants performed a computerized decision-making task in the context of finding the best health care plan. Data included quantitative decision-making performance indicators and surveys examining previous history with purchasing insurance. Participants used a colored info-vis ES or a table (no ES) to perform the decision task. Task difficulty was manipulated by increasing the number of selection criteria used to make an accurate decision. A repeated measures analysis was performed to examine differences between the two table designs.

**Results:**

Twenty-three older adults between the ages of 66 and 80 completed the study. There was a main effect for accuracy such that older adults made more accurate decisions in the color info-vis condition than the table condition. In the low difficulty condition, participants were more successful at choosing the correct answer when the question was about the gap coverage attribute in the info-vis condition. Participants also made significantly faster decisions in the info-vis condition than in the table condition.

**Conclusions:**

Reducing the working memory demand of the task through the use of an ES can improve decision accuracy, especially when selection criteria is only focused on a single attribute of the insurance plan.

## Introduction

### Older Adults’ Difficulties With the Medicare Website

A usability evaluation of the Medicare website revealed that older adults were unable to successfully choose a prescription drug plan for a given medication regimen [1]. Example problems highlighted in the evaluation included general difficulties navigating the site, frustration, and the inability to locate desired information. Compared to younger age groups, older adults have less success obtaining Internet health information [2]. Insurance and medical jargon (eg, “gap coverage”, “drug sharing”, etc) may have further exacerbated the difficulties. Even without time constraints, difficulties in identifying the best plan in a demanding environment can lead users to select a plan that does not provide adequate medical coverage or is more expensive than other available options [3].

The number of prescription drug plans presented to users can cause severe problems, especially when users attempt to simultaneously compare choices across different criteria. Simply increasing the number of available Medicare drug plans from 3 to 9 is associated with poorer decision outcomes that can negatively affect quality of life, quality of care, and overall health [4]. Poorer decision outcomes result from the increased working memory demands associated with comparing a larger number of plans. Trying to make optimal decisions in the face of uncertainty with a large amount of inputs is a working memory demanding task (see Multimedia Appendix 1). In particular, comparing plans across different criteria and calculating costs (steps 5-9) illustrates the working memory-intensive nature of selecting an appropriate prescription drug plan. The working memory-intensive nature of the task combined with older adults’ reduced capacity for working memory [5] result in a reduced ability to discern between plan costs as the number of plans increase compared to their higher cognitive functioning counterparts [6]. In sum, choosing an optimal health plan is a task that places large demands on working memory and attention and can result in negative consequences if decision-making performance suffers. A decision aid designed to redirect task demands from working memory to an external memory aid may facilitate optimal decision-making in older adults.

### The Importance of Working Memory in Decision-Making

Working memory capacity refers to the amount of information one can temporarily store and manipulate at any given time [7]. If a task’s working memory demand exceeds one’s working memory capacity, then task performance declines. This capacity limit is central to one’s ability to process information and make a decision. The information processing model of decision-making [8] is a useful tool for understanding working memory demands at each step of the decision-making process. According to the model, information must first be perceived then selectively attended to by the decision maker. Next, the decision maker generates hypotheses about specific outcomes and selects a decision or response. Finally, the decision maker implements the response and compares the outcome to the initial set of hypotheses. Each step of this model as it relates to choosing a prescription drug plan is discussed in further detail below.

Attentional limitations force the user to filter cues relevant to the decision goal from the irrelevant cues by selectively attending to only some of the information present. Choosing a prescription drug plan on the basis of cost first requires that the decider perceive the appropriate cues (eg, monthly premiums, coverage in the gap), while ignoring irrelevant cues (eg, Medicare ID numbers or contact information). Cues are selected based on their diagnosticity (amount of information the cue provides), reliability (trustworthiness of information), and salience (physical properties such as volume, color, and shape). After cues are selected for further processing, they are compared to other information to form a meaningful interpretation of the state of the system.

After selectively attending to appropriate cues, the information is manipulated in working memory where hypotheses or potential outcomes are generated (eg, plans with a low monthly premium and low deductibles have less coverage). Choosing a prescription drug plan requires several hypotheses for each plan; one for cost and the effect on personal budget, one for satisfaction, etc. Here, working memory limitations prevent a truly exhaustive comparison. The next step involves integrating the outcomes and action selection. At this stage, the decision maker tries to determine which option will produce an outcome that best meets the goal. If a plan is selected for its low monthly premium, but also has a low satisfaction rating, the decision maker has to consider the potential implications of both attributes together. This step is highly error-prone because working memory capacity limits the number of comparisons that can be made simultaneously. When an action is selected and carried out (a decision is made), the outcome is monitored and evaluated against new cues or information, and new hypotheses about the state of the system are formed. Here again, working memory capacity limits the amount of new information that can be selected and then compared to the current state of the system, potentially harming the ability to select the best plan.

### Effects of Aging on Decision Making

Older adults’ reduced working memory capacity [5] limits the number of integration and comparison tasks that can be made at a given time and thus will affect their ability to make optimal decisions [9]. Age-related limitations may force older adults to rely more heavily on heuristic-based decision making (ie, “rules of thumb” or cognitive shortcuts used to make decisions quickly with little effort) [10], which may not always lead to an optimal decision. Although older adults are sometimes successful in adapting their strategies to meet task demands, they tend to perform worse on the task of integrating information (comparing more than two pieces of information) and extracting information (finding one piece of information) [11]. For example, comparing information that is presented in different units (eg, monetary units and satisfaction ratings) could make a task more difficult for older adults [12,13]. Indeed, when choosing a prescription drug plan, one must compare multiple cost values and multiple satisfaction ratings across many different plans.

Older adults also tend to commit more errors and have more difficulty comprehending information than younger adults when the task requires integrating information [12] among many choices [13]. One way to reduce errors, besides reducing the number of possible choices, is to include specific visual aids that guide attention toward more relevant choices and help eliminate the need to hold information about less relevant choices in working memory.

Although working memory limits the amount of information used to make optimal decisions, information that shares similar perceptual or semantic features may be grouped together into object-like “chunks” or visual clusters that enable pattern recognition [14-16], effectively overcoming some working memory limitations. Information may be chunked together based on color, shape, meaning, spatial proximity, or other properties (eg, Gestalt principles) [17] preattentively or automatically (without the need to selectively attend to each cue individually). This perceptual integration process may help facilitate processing of more information with less effort.

Aids that reduce working memory demands are called environmental supports (ES) [18]. ESs often utilize perceptual integration principles to improve task performance for older adults by reducing task demands or supporting the use of existing resources [19,20]. Several studies with younger adults have shown that providing an ES reduces working memory demand by facilitating visual search and automatic perceptual processing of information from graphs [15] by visually integrating related information into meaningful chunks using color [21]. Ratwani et al [15] theorized that when information within a graph is organized into visual “clusters”, less effort is needed to group similar information together, which reduces the working memory demand of the task; the user can focus attention on the differences between the groups, rather than first actively integrating information into clusters.

Reducing the need for effortful comparison of information will allow the user to allocate more resources to later steps in the decision-making process, which could result in more thorough outcome predictions (eg, how a plan might affect finances) and appropriate action selections (eg, choosing one plan over another) [15]. Older adults may benefit from a decision aid designed to shift task demands from working memory to an external memory aid [22], where it can be perceived by the relatively age-insensitive preattentive visual perceptual system [23]. For example, an ordered brightness scale allows people to make comparisons between choices without having to process a number and assign it meaning before serially moving on to the next choice [24]. Instead, meaning is automatically processed using perceptual features (eg, darker green may represent a higher number than a lighter green, the scale is based on the color density). Additionally, it is much faster to search for a color singleton than to find a number target [25]. This suggests a promising avenue of providing an ES-based decision-making aid: shifting the working memory burden to the perceptual processing system by eliminating the need to comprehend and compare each option semantically, and instead comparing the information perceptually.

### Objective

The objective of the current study was to extend Lohse's [21] and Ratwani et al's [15] findings to the design of an information visualization (info-vis) aid in order to examine whether older adult decision-making performance can be enhanced by the use of graphical decision aids designed to reduce working memory demands. Reducing working memory demands was expected to lessen reliance on less accurate heuristic strategies and improve decision quality. Decision quality was measured by how well the choice met the criterion in the question. The assumption was that when the decision-making task is reduced from cognitively complex to relatively easy, decision makers would not need to rely on heuristics and would consider all relevant information. Specifically, we predict an interaction between decision aid and task difficulty such that the more difficult task decisions will be benefitted most by the info-vis aid. Because the info-vis aid was designed to reduce working memory demands of the task, the performance gains will be greater for difficult tasks that require more working memory resources.

## Methods

### Participants

There were 23 older participants ages 65-80 that were recruited through an existing database of volunteers in the surrounding community. Older adults received US $14 in compensation for participating. Color-blindness and the inability to read a computer screen were the only exclusion criteria.

### Design

The study was a 2 (decision aid: table, color info-vis) x 2 (task difficulty: low, high) repeated measures design, with decision aid as the between-subjects variable and task difficulty as the within-subjects variable. Therefore, each participant was randomly assigned to one of the decision aid conditions and completed trials at both levels of task difficulty. Participants made decisions on a total of 20 trials. The trials were organized around 4 blocks of 5 questions per block. A randomized blocked design was utilized for questions of varying task difficulty. The questions within each block were also randomly presented. Dependent measures included decision accuracy (sum score of number correct), decision quality (sum score of scaled decision ratings for the high difficulty questions), and decision task time (in seconds).

### Materials

Demographic information, health information, insurance experience, technology experience, a working memory measurement, and an exit survey were collected from each subject. A blocked design allowed us to administer the subjective workload measure (NASA-TLX) at the end of each block for each level of task difficulty and working memory demand. Participants used PC-compatible computers and wore headphones during the experiment. The experiment was programmed using E-prime (version 1.1).

### Task

#### Decision Task

The decision task utilized a computerized decision-making paradigm presented in the context of choosing the best health care plan based on given criteria. All participants were assigned to one of the two decision aid conditions and performed tasks at both levels of difficulty. A standardized format was used so that the question, plan data, and choice set always appeared in the same location for each trial. The question was located at the top of the screen, with the decision aid below it.

#### Decision Aids

**Figure 1:**
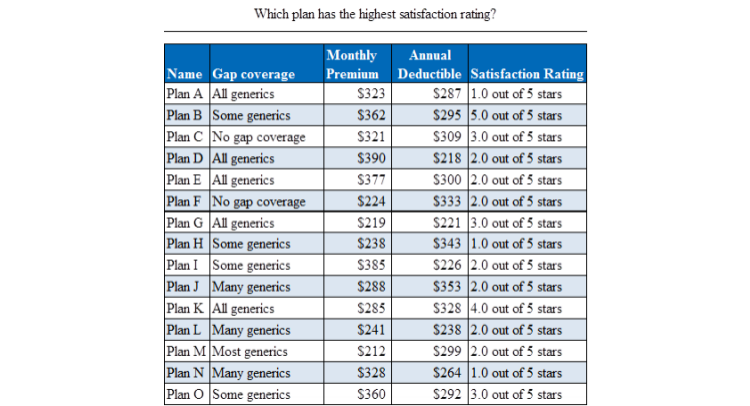
Example layout of a low difficulty decision task in the table condition. Fifteen plan options are shown with four plan attributes (gap coverage, monthly premium, annual deductible, and satisfaction rating).

The table condition was a replica of the table found on the Medicare website (as shown in 2010). The table included a row for each of the fifteen prescription drug plans and columns for four of the plan’s attributes (Figure 1 shows this). In 2014 and 2015, an average of eighteen Medicare Advantage plans were available to enrollees [26]; therefore fifteen plans are representative of a typical Medicare plan selection task.

**Figure 2:**
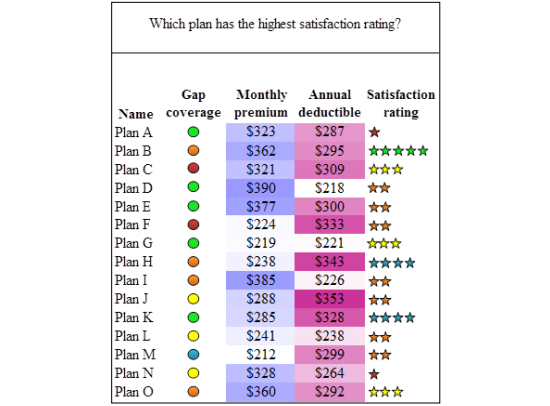
Color information visualization (color info-vis).

The info-vis condition was created by adding graphics instead of (or in addition to) text to represent specific attributes. Visualization tools are able to help users interpret large sets of information quickly and efficiently [27]. A single info-vis (Figure 2 shows this) was created utilizing well-accepted display design principles (eg, proximity compatibility principle, color gradients, pictorial representations, and redundancy) [28,29]. The info-vis used in this study was specifically designed to alleviate the working memory intensive parts of the task by converting them into easier perceptual tasks using a color manipulation. Color info-vis uses multi-colored scales (heat map color scale) to replace the categorical gap coverage text. The same multi-colored scale was used in the stars that replace the number scales for satisfaction ratings. The colors highlight the relevant information within each attribute and create fewer mental comparisons for the user. Multi-colored scales can facilitate identification tasks—where one has to select a target value represented by a color (eg, identify the plans that have gap coverage level of all generics—represented by the color green), and in cases where a particular absolute value (ie, all generics) is more important than a relative value (ie, the plan with the lowest amount of coverage) [24]. In the current study, the multi-colored scale was used to represent the five specific categories of both gap coverage and satisfaction ratings and these categories were absolute, not relative to one another (eg, “all generics” was always the highest level of gap coverage, but “some” or “many” generics are not proportionate to each other).

Brightness ordered scales (same color is used, but lightest color gradient is the lowest value and the darkest color is the highest value) were added to dollar amounts in both the monthly premium and annual deductible columns. Brightness ordered scales have been shown to be superior for comparisons of relative value [24] where all values are compared to one another (eg, which plan has the lowest or highest monthly premium). These color manipulations were added to facilitate more perceptual comparisons rather than effortful cognitive comparisons, thus reducing working memory demand. Each attribute in the display layout was grouped close together in perceptual space to make comparisons easier (ie, proximity compatibility principle) [29]. Pilot testing on a younger adult sample found that color info-vis does minimize working memory demand. Participants made more accurate decisions with a color info-vis than size or no info-vis. Size info-vis used pie and bar graphs to indicate differences between drug plans for each criterion. The pilot testing followed a similar procedure except that an auditory n-back task was used to constrain younger adults’ working memory capacity. The secondary task simulated the limitations on older adults’ cognitive abilities.

#### Task Difficulty

Task difficulty was manipulated by varying the number of plan attributes that must be considered in order to accurately complete the task. In the low difficulty condition, participants selected a plan based on one attribute (eg, Which plan has the lowest monthly premium?). The high difficulty condition required the participant to select a plan by integrating and comparing three attributes of each plan (eg, Which plan has the lowest monthly premium, highest gap coverage, and highest satisfaction rating?).

For both conditions, the data were structured so that only one plan best met all of the criteria in the question. This manipulation required participants to make a compensatory decision (choosing the best plan by evaluating alternatives along with the required selection criteria) and use an analytical decision strategy in order to select the best answer [30], thus, using heuristics would not lead to the optimal answer choice. Participants in the low difficulty condition only had to compare the values for a single attribute. In the current info-vis table, each attribute is integrated with graphics that makes identifying the optimal choice for each attribute less cognitively demanding. he low difficulty condition is practically useful because single-attribute decision making is a common heuristic in naturalistic decision-making [30] and establishes a baseline of performance on which other conditions can be compared against. Analysis of a single-attribute decision will answer whether the graphical representation (info-vis) can also affect the efficiency and accuracy of identifying the best option based on a single attribute. In the high difficulty condition, participants needed to rank the values for each attribute and add them together to identify the best plan. The high difficulty condition approximates rational decision-making techniques that consider larger amounts of information and require greater cognitive demands.

### Procedure

Experimental sessions were administered in groups of 1 to 4 participants; however, each participant worked independently. After providing informed consent, participants completed a paper and pencil working memory ability test, the Reverse Digit Span [31], before moving on to the computerized portion of the task.

The terms used in the decision task (eg, gap coverage) were defined by the experimenter and also presented visually on the screen. Participants first completed a series of practice questions that introduced low and high difficulty problems of the decision-making task. Participants chose an answer by pressing the letter on the keyboard that corresponded with the selected plan (eg, participants pressed the “A” key to select Plan A). At the end of the practice task, a screen prompted users to fill out the NASA-TLX survey. Participants then began the recorded trials. Each recorded block involved the same procedure as the practice block. At the conclusion of the task, participants completed the demographics and health survey, a technology experience survey, an insurance purchasing experience questionnaire, and an exit survey.

## Results

### Participants

There were 23 older adults (12 female) between the ages of 66 and 80 (mean, M, 72.4, SD 3.73) that participated in this study. Many of the participants indicated they had previous experience purchasing insurance (ie, 19 out of 23 participants, 83%, bought Medicare plans, 14 out of 23, 61%, bought prescription drug insurance, and 20 out of 23, 87%, bought health insurance). No significant differences (*P*>.05) were found between decision aid groups on computer experience, health, insurance purchasing experience, working memory, or age. Therefore, all subjects were included in the following analyses.

### Decision Accuracy

A 2 (decision aid) x 2 (difficulty) repeated measures analysis of variance (ANOVA) revealed a significant main effect of task difficulty on decision accuracy (*F*_1, 21_=39.88, *P*<.001, *η*^2^=.65). Participants performed the decision task more accurately in the low difficulty condition (M 8.87, SD 1.39) than in the high difficulty condition (M 6.30, SD 2.05). There was a significant main effect of decision aid (*F*_1, 21_=3.81, *P*=.03, *η*_p_^2^=.15), which confirms the hypothesis that older adults would perform significantly better in the color info-vis condition (M 8.13, SD 1.21) than the table condition (M 7.00, SD 1.55). 

The interaction between task difficulty and decision aid was not significant (*F*_1, 21_=.829, *P*=.19, *η*_p_^2^=.04).

**Figure 3:**
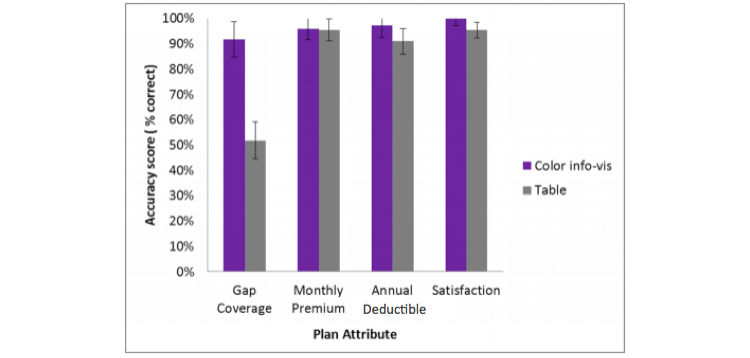
Percent accuracy on low difficulty tasks by plan attribute and decision aid. Error bars represent standard error of the mean.

For the low difficulty decision tasks, participants were asked to find a plan that best meets the single criterion (one attribute, eg, satisfaction rating). Therefore, we can analyze performance for each attribute (gap coverage, monthly premium, annual deductible, and satisfaction rating) individually to examine why participants were more accurate in the info-vis condition than in the table condition. Because the high difficulty condition involves locating a plan that meets several criteria, we can only assess the source of the main effect of decision aid in the low difficulty condition. The low difficulty condition data were analyzed using a 2 (decision aid) x 4 (plan attribute) mixed measures ANOVA. Main effects of attribute type (*F*_1.72, 36.11_=15.61, *P*<.001, *η*_p_^2^=.43) and decision aid (*F*_1, 21_=7.10, *P*=.02, *η*_p_^2^=.25) were qualified by a significant interaction between plan attribute and decision aid (*F*_1.72, 36.11_=8.81, *P*=.001, *η*_p_^2^=.30). Figure 3 shows this. Participants were better able to accurately answer questions about the gap coverage attribute in the color info-vis condition (M 91.7%, SD 20.77%) than in the table condition (M 51.73%, SD 27.51%). This difference is the source of the main effect of decision aid on accuracy.

### Decision Task Time

**Figure 4:**
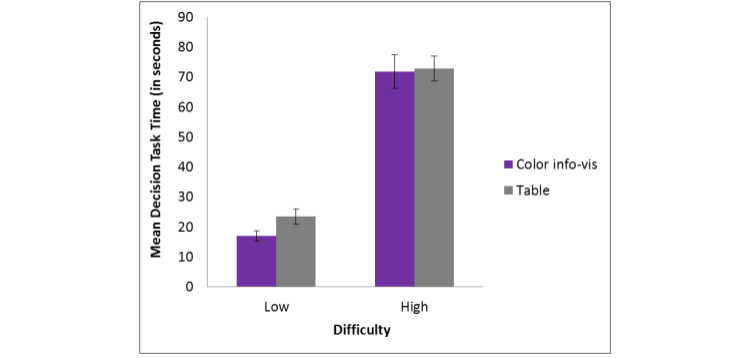
Decision task time by decision aid for low and high difficulty tasks. Error bars represent standard error of the mean.

A 2 (decision aid) x 2 (difficulty) repeated measures ANOVA was run to assess decision task time (in seconds, s) and revealed a significant main effect of difficulty (*F*_1, 21_=155.73, *P*<001, *η*_p_^2^=.88), such that participants were faster in the low difficulty condition (M 20.07 s, SD 7.78 s) than in the high difficulty condition (M 70.69 s, SD 20.92 s). Figure 4 shows this. There was no significant main effect of decision aid (*F*_1, 21_=1.07, *P*=.31, *η*_p_^2^=.05) on task time, nor was there an interaction between decision aid and difficulty (*F*_1, 21_=.081, *P*=.78, *η*_p_^2^=.01).

**Figure 5:**
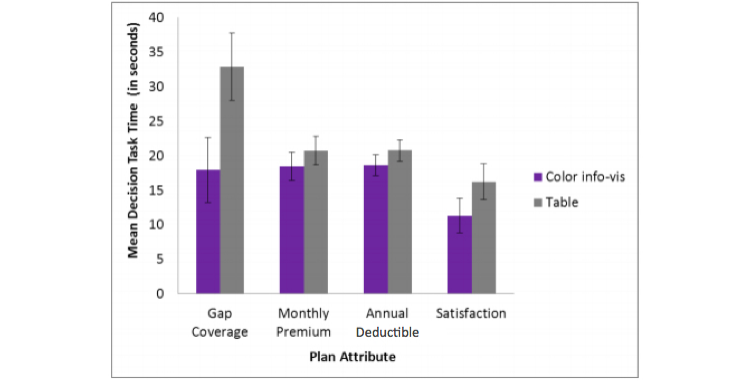
Mean decision time (in seconds) by plan attribute and decision aid for the low difficulty condition. Error bars represent standard error of the mean.

A 2 (decision aid) x 4 (plan attribute) repeated measures ANOVA on decision time (in s) was run to look for evidence of a speed-accuracy trade-off that might explain the effect of decision aid on accuracy with gap coverage questions. We can only assess the source of the main effect in the low difficulty condition because the high difficulty condition involves locating a plan that meets several criteria. The analysis revealed a significant main effect of decision aid (*F*_1, 21_=4.5, *P*=.046, *η*_p_^2^=.18) and a significant main effect of plan attribute (*F*_6.8, 37.68_=6.82, *P*=.004, *η*_p_^2^=.25), but not a significant interaction between decision aid and attribute (*P*=.08). Participants spent more time answering the gap coverage questions than the other attributes and more time answering questions about this attribute in the table condition than in the color info-vis condition (Figure 5 shows this).

Participants answered the decision task significantly faster in the color info-vis condition (M 16.93 s, SD 5.95 s) than in the table condition (M 23.5 s, SD 8.35 s). Questions about the satisfaction rating attribute (M 13.69 s, SD 8.81 s) took significantly less time than the annual deductible (M 19.64 s, SD 5.22 s), gap coverage (M 25.41 s, SD 17.66 s), and monthly premium (M 19.56 s, SD 6.86 s). This indicates that there was not a speed-accuracy trade-off that would explain significantly lower accuracy for gap coverage questions in the table condition versus the color info-vis condition.

### Decision Quality

For each high difficulty question, the plan data were created so that only one option met all of the criteria presented in the question during each trial. The other plan options met only 0, 1, or 2 of the 3 possible criterion. Choosing the correct plan assumes that each criterion was used in the assessment. Thus, a maximum score of 3 is possible for each question and represents the best answer. A minimum score of 0 indicates that the plan chosen met none of the criteria in the question. These points were added together to compute a total decision quality score for the high difficulty questions. For the computed score, the maximum score was 30 points (3 x 10 questions) and the minimum score was 0 points.

An independent samples *t* test was conducted between decision aid conditions on decision quality score and revealed that quality did not differ significantly by decision aid (*t*=.7, *P*=.49). A one-tailed significance test did not change the effect of the decision aid variable on decision quality.

### Subjective Workload

Subjective workload ratings were assessed by conducting a 2 (decision aid) x 2 (difficulty) repeated measures ANOVA. A significant main effect of difficulty (*F*_1, 21_=74.2, *P*<.001, *η*_p_^2^=.78) was revealed in the expected direction. This was a manipulation check for difficulty and indicates a successful manipulation because participants rated the high difficulty tasks as significantly more difficult (M 58.63, SE 3.57) than the low difficulty tasks (M 35.35, SE 2.99). There was no main effect of decision aid (*F*_1, 21_=1.5, *P*=.23, *η*_p_^2^=.07), nor an interaction effect of decision aid and difficulty (*F*_1, 21_=.06, *P*=.82, *η*_p_^2^=.003).

## Discussion

### Principal Findings

This study examined whether color info-vis can be used as a decision support for older adults making complex decisions. Previous research has shown that older adults exhibit difficulty in choosing a prescription drug plan on the Medicare website, possibly because of a combination of usability issues and normative changes in cognitive abilities such as reduced working memory capacity [1]. It was hypothesized that older adults would perform better (higher accuracy and quality) in the color info-vis condition than in the table condition for both high and low difficulty tasks. Our results show that accuracy was significantly higher in the color info-vis condition (shifting processing burden from cognitive resources to perceptual resources) than in the table condition, indicating that older adults did not use heuristics, but instead an analytical decision-making strategy.

If older adults did not choose the best plan option, they were able to select a plan that was “good enough” in quality regardless of the decision aid. This finding is consistent with the finding that older adults are more likely to use heuristic strategies at a lower level of working memory demand than younger adults and that they can use heuristics successfully [10].

It was hypothesized that performance in the difficult task condition would benefit most from the info-vis display. However, the interaction between task difficulty and decision aid was not significant. The lack of an effect of condition on accuracy in the high difficulty tasks indicates that relying on perceptual capacities cannot fully accommodate age-related declines in cognitive capacities (color info-vis condition). Although the color info-vis may have been successful in reducing the working memory demand for comparing plans on a single attribute (low difficulty task), the info-vis did little to support integration of more than one attribute (ie, the three attributes required in the high difficulty tasks). That is, the info-vis display used color to reduce working memory demands when making decisions within each attribute, but the table did not facilitate information integration or show relationships among different attributes. This could also account for the finding that the type of aid did not influence perceived workload. Future research should evaluate ways to support more complex decision-making tasks where multiple attributes must be integrated and compared through info-vis (eg, configural displays).

In the graph reading literature, a low difficulty condition is generally termed an extraction task because the user is asked to find a specific bit of information (eg, what is Plan B’s monthly premium amount), rather than perform a comparison of one attribute among many options (eg, which plan has the lowest monthly premium) as in this study. This may be why there was an effect in the low difficulty condition that is not consistently found in other studies within the graph reading literature [15].

In the low difficulty condition, older adults were much more successful at choosing the correct answer when the question was about the gap coverage attribute. This finding is interesting for a number of reasons. First, although the performance boost in the gap coverage attribute is selective, it could be due to ceiling effects in the accuracy data, and floor effects in task time data. The gap coverage attribute had the most room for improvement among the other attributes in both accuracy and task time. In the table condition, accuracy for the monthly premium, annual deductible, and satisfaction ratings attributes all approached near optimal levels of accuracy, while gap coverage yielded less accurate responses. There was similar room for improvement in the task time data such that task times in the table condition for monthly premium, annual deductible, and satisfaction rating were around 20 s compared to approximately 33 s for the gap coverage decision. Second, the user had to remember what each of the colors meant or had to refer to the legend, which on the surface appears to increase working memory demand. However, in the table condition, gap coverage had to be evaluated based on textual values (eg, all generics vs some generics). This requires reading and comprehension of the text, rather than a less working memory demanding visual search for a target color [25]. Third, previous literature has suggested that numeracy (ability to process numerical information) and processing speed (or how fast one can process information and perform tasks without focused attention) are responsible for performance differences with a large dataset (24 plan options) [13]. Using color comparisons rather than numerical comparisons may be a good option for those who do not have high numeracy abilities, working memory abilities, and those with slower processing speed.

Whether or not the use of color is in fact allowing the user to make faster, less demanding comparisons might be a question that can be answered using eye-tracking data. For example, recording fixation durations and plotting saccadic amplitude could help answer the question of whether color is facilitating a less cognitively demanding search [32]. Long fixation durations might indicate focal vision, which is indicative of selective attention, while short saccades indicate a scanning behavior akin to ambient vision or more automatic (preattentive) processing.

Due to the design of the study, it is difficult to conclude whether the selective benefits of the info-vis display reflect limitations of color integration in displays for older adults, or the ways in which color was implemented in our info-vis condition. If the results reflect the latter, this could explain why significant improvements were only observed for a single attribute (gap coverage). The task improvement could be due to the substitution of textual data for the visual color scale. This change, unlike the other attributes, represents a transformation of the data from textual jargon, to a familiar color scale, which removes the need for knowledge of the specific meanings of each category. In the other three attributes, color was used in addition to the numeric and textual information (especially in the monthly premium and annual deductible attributes). Therefore, the transformation from textual information to the heat mapping color scale in the gap coverage attribute may be more beneficial for older adults than searching through data that is overlaid with color saturations which suggests rankings (that still contain numerical information that older adults could choose to use in their comparison rather than solely relying on the color saturations). Although the benefits of color info-vis are well established among younger adults, there may be limitations of benefits in older adults. Because older adults have the greatest amount of difficulty with information integration tasks [11], a configural display that illustrates relationships among different attributes using color and shape could have further boosted older adults’ decision performance. Future research should examine how perceptual manipulations (eg, color, size, and shape) interact together and whether high difficulty comparisons and integration tasks can be simplified. This study did not examine the effects of size and color together or how these manipulations can improve specific types of data (eg, categorical vs interval). Another limitation of this study was the small sample size in each decision aid condition. As such, the results may provide low power to detect aid-related effects on performance.

### Conclusions

Reducing the working memory demand of the task through the use of an ES can improve decision accuracy in certain cases. The results of this study indicate that color info-vis may be a viable ES for older decision makers for comparison tasks. Additionally, decision-making based on a single attribute can lead to better selection of drug plans for older adults. Instead of presenting all attributes at once for users to compare and contrast, each attribute could be presented individually and feature ES to reduce working memory demand. Faceted information retrieval is a filtering system that allows users to search along a specific feature or attribute [33]. Many search engines and retail websites utilize faceted search for improved navigation. This search method would yield a more manageable set of drug plans to choose from because less desirable plans would be filtered out with each attribute. Further research is needed to examine whether the use of color info-vis for each attribute could improve the quality of selected drug plans and reduce the time spent identifying the optimal choice compared to the current complex table design.

## Appendix

Multimedia Appendix 1 Task analysis for choosing a prescription drug plan from the Medicare website.

## Abbreviations

ANOVA: analysis of variance
color info-vis: color information visualization
ES: environmental supports
info-vis: information visualization
M: mean
s: seconds

## References

[1] Czaja SJ, Sharit J, Nair SN (2008). Usability of the Medicare health web site. JAMA.

[2] Agree EM, King AC, Castro CM, Wiley A, Borzekowski Dina L G (2015). “It's got to be on this page”: Age and cognitive style in a study of online health information seeking. J Med Internet Res.

[3] Hsu J, Fung V, Price M, Huang J, Brand R, Hui R, Fireman B, Newhouse JP (2008). Medicare beneficiaries' knowledge of Part D prescription drug program benefits and responses to drug costs. JAMA.

[4] Hanoch Y, Wood S, Barnes A, Liu PJ, Rice T (2011). Choosing the right medicare prescription drug plan: The effect of age, strategy selection, and choice set size. Health Psychol.

[5] Salthouse TA (1990). Working memory as a processing resource in cognitive aging. Developmental Review.

[6] McWilliams JM, Afendulis CC, McGuire TG, Landon BE (2011). Complex Medicare advantage choices may overwhelm seniors--especially those with impaired decision making. Health Aff (Millwood).

[7] Baddeley A (1987). Working memory.

[8] Wickens C (1992). Engineering psychology and human performance.

[9] Mata R, Schooler LJ, Rieskamp J (2007). The aging decision maker: Cognitive aging and the adaptive selection of decision strategies. Psychol Aging.

[10] Chen Y, Sun Y (2003). Age differences in financial decision-making: Using simple heuristics. Educational Gerontology.

[11] Finucane ML, Slovic P, Hibbard JH, Peters E, Mertz CK, MacGregor DG (2002). Aging and decision-making competence: An analysis of comprehension and consistency skills in older versus younger adults considering health-plan options. J. Behav. Decis. Making.

[12] Finucane ML, Mertz CK, Slovic P, Schmidt ES (2005). Task complexity and older adults' decision-making competence. Psychol Aging.

[13] Tanius BE, Wood S, Hanoch Y, Rice T (2009). Judgment and Decision Making.

[14] Miller GA (1956). The magical number seven plus or minus two: Some limits on our capacity for processing information. Psychol Rev.

[15] Ratwani RM, Trafton JG, Boehm-Davis DA (2008). Thinking graphically: Connecting vision and cognition during graph comprehension. J Exp Psychol Appl.

[16] Grierson HJ, Corney JR, Hatcher GD (2015). Using visual representations for the searching and browsing of large, complex, multimedia data sets. International Journal of Information Management.

[17] Pomerantz JR, Pristach EA (1989). Emergent features, attention, and perceptual glue in visual form perception. J Exp Psychol Hum Percept Perform.

[18] Klix F, Hagendorf H (1986). A functional account of age differences in memory. Human memory and cognitive capabilities: Mechanisms and performances: symposium in memoriam Hermann Ebbinghaus - 1885 - Berlin Humboldt University - 1985.

[19] Morrow DG, Rogers WA (2008). Environmental support: An integrative framework. Hum Factors.

[20] Sharit J, Czaja SJ, Nair S, Lee CC (2003). Effects of age, speech rate, and environmental support in using telephone voice menu systems. Hum Factors.

[21] Lohse GL (1997). The role of working memory on graphical information processing. Behaviour & Information Technology.

[22] Pak R, Price MM, Thatcher J (2009). Age-sensitive design of online health information: Comparative usability study. J Med Internet Res.

[23] Plude DJ, Doussard-Roosevelt JA (1989). Aging, selective attention, and feature integration. Psychol Aging.

[24] Breslow LA, Ratwani RM, Trafton JG (2009). Cognitive models of the influence of color scale on data visualization tasks. Hum Factors.

[25] Treisman A (1982). Perceptual grouping and attention in visual search for features and for objects. J Exp Psychol Hum Percept Perform.

[26] Jacobson G, Gold M, Damico A, Neuman T, Casillas G (2015). Medicare advantage 2016 data spotlight: Overview of plan changes.

[27] Arden-Close EJ, Smith E, Bradbury K, Morrison L, Dennison L, Michaelides D, Yardley L (2015). A visualization tool to analyse usage of web-based interventions: The example of positive online weight reduction (POWeR). JMIR Human Factors.

[28] Galitz WO (2007). The essential guide to user interface design: An introduction to GUI design principles and techniques.

[29] Wickens CD, Carswell CM (1995). The proximity compatibility principle: Its psychological foundation and relevance to display design. hum factors.

[30] Payne JW (1976). Task complexity and contingent processing in decision making: An information search and protocol analysis. Organizational Behavior and Human Performance.

[31] Wechsler D (1997). Wais-3 administration and scoring manual.

[32] Velichkovsky BM, Joos M, Helmert JR, Pannasch S (2005). Two visual systems and their eye movements: Evidence from static and dynamic scene perception.

[33] Tunkelang D (2009). Faceted search. Synthesis lectures on information concepts, retrieval, and services.

