# Supplementary material for: Effects of Information Visualization on Older Adults’ Decision-Making Performance in a Medicare Plan Selection Task: A Comparative Usability Study
Source: JMIR Hum Factors. 2016 Jun 1;3(1):e16. doi: 10.2196/humanfactors.5106 (PMC4909975; doi:10.2196/humanfactors.5106)
Supplement: Multimedia Appendix 1 [file humanfactors_v3i1e16_app1.pdf]

APPENDIX A

Task Analysis for Choosing a Prescription Drug Plan from the Medicare.gov Website

Task #	Task	Task/Knowledge Requirements*	Feedback	Potential Problems
1.0	**Observe the table**			
1.1	Read each of the headings	Reading comprehension[7]	None	Does not understand jargon
1.1.1	Click on "What is this?" if unsure what the heading means	Visual search[8]	Small window appears	Fails to see link
1.1.2	Read the definition	Reading comprehension[7]	None	Does not understand definition
1.1.3	Press the x button to close the window	Declarative knowledge[4]	Small window closes	Unable to figure out how to close window and return to previous window
2.0	**Find total yearly fixed cost**			
2.1	Locate plan with cheapest monthly premium	Visual search[8]	None	Forget which plan had the lowest amount
2.1.1	Extract the monthly premium amount	Visual search[8]	None	
2.2	Multiply premium by 12 months on paper	Numerical computation[6]; Working memory[9]	None	May miscalculate amount
2.3	Locate the annual deductible	Visual search[8]; Working memory[9]	None	Forget which plan to use
2.4	Add deductible to the premium total	Numerical computation[6]; Working memory[9]	None	Forget which value was the premium total
3.0	**Calculate out-of-pocket expenses**			
3.1	List current drug costs	Declarative knowledge[4]	None	May not remember all costs; may miscalculate amount
3.1.2	Add up the monthly cost of your drugs	Numerical computation[6];	None	May miscalculate cost

		Working memory[9]		
3.1.3	Multiply monthly drug cost by 12 months	Numerical computation[6]; Working memory[9]	None	May miscalculate cost; drug costs may differ over the year
3.2	Calculate expenses outside of the gap	Numerical computation[6]; Working memory[9]	None	
3.2.1	Find the donut hole (gap in coverage) amount	Abstract reasoning[1]; Reading comprehension[7]	None	May not understand jargon or meaning
3.2.2	Click on the "what is this?" link in the "coverage in the gap" column	Reading comprehension[7]	Small window appears	Fails to see link; may not understand jargon
3.2.3	Read explanation	Reading comprehension[7]	None	May not understand explanation
3.2.4	Extract the donut hole amount	Visual search[8]	None	May not identify correct #
3.2.5	Subtract total yearly drug cost from the donut hole amount	Numerical computation[6]; Working memory[9]	None	Forget which amount was the total yearly drug cost
3.2.6	Divide that number by monthly cost of drugs to determine months of no coverage	Numerical computation[6]; Working memory[9]	None	Forget the monthly cost of drugs; May not understand how to do this task
3.2.7	Round that number up	Abstract reasoning[1]	None	
3.2.8	Multiply number of months without coverage by monthly cost of drugs (cost w/o using insurance)	Numerical computation[6]; Working memory[9]	None	Forget the monthly cost or calculated number of months without coverage
3.2.9	Add this to number 3.1.3	Numerical computation[6]; Working memory[9]	None	May forget the monthly drug cost before the donut hole
3.3	Calculate drug sharing costs		None	May not understand jargon

3.3.1	Click on the plan name to get to the detail page	Good visual acuity[5]; Attentional control[2]; Abstract reasoning[1]	Web page displays plan details	Fails to see or understand link
3.3.2	Scroll down to find the drug cost sharing box	Visual search[8]	Page moves with scrolling action	Fails to scroll to see more details
3.3.3	Read tier cost information	Reading comprehension[7]	None	May not understand jargon
3.3.4	Contact the drug plan to find out what tier your drugs are in (recommended)	Declarative knowledge[4]	Speak to representative	Unable to do this task independently
3.3.5	Calculate cost of drugs by multiplying the tier cost by number of drugs	Numerical computation[6]; Working memory[9]	None	Forget which drugs are in what tier or how much each tier was worth; Forget which plan is being evaluated
3.3.6	Add up totals	Numerical computation[6]; Working memory[9]	None	May miscalculate totals; Forget to add in a total
3.3.7	Add this number from 3.2.9	Numerical computation[6]; Working memory[9]	None	Forget amount from 3.2.9
4.0	**Consider customer satisfaction ratings**			
4.1	Find the column for summary ratings	Visual search[8]	None	
4.1.1	Extract summary rating	Visual search[8]	None	
4.1.2	Click on the plan name to get to the detail page	Abstract reasoning[1]; Good visual acuity[5]	Web page displays plan details	Fail to realize detailed information exists
4.1.3	Find the box that contains all of the rating categories	Visual search[8]	None	Fail to scroll down
4.1.4	Read the label and definition of each rating category	Reading comprehension[7]	None	May not understand jargon
4.1.5	Compare the	Comparison[3],	None	May not

	number of stars for each category	Working memory[9]		understand stars
5.0	**Compare yearly cost and monthly cost to current budget**	Comparison[3], Working memory[9]	None	May forget monthly or yearly totals
6.0	**Repeat steps 2.0-4.1.2 for another plan**		None	May forget comparable values from prior plan
7.0	**Compare plan to another plan**			May forget comparable values from prior plan
7.1	Compare yearly fixed cost	Comparison[3], Working memory	None	May forget comparable values from prior plan
7.2	Compare out-of-pocket expenses	Comparison, Working memory[9]	None	May forget comparable values from prior plan
7.3	Compare nationwide coverage	Comparison[3], Working memory[9]	None	May forget comparable values from prior plan
7.4	Compare satisfaction ratings	Comparison[3], Working memory[9]	None	May forget comparable values from prior plan
**8.0**	**Repeat steps 2.0-7.4 for another plan**		None	May forget comparable values from prior plan
**9.0**	**Compare against all plans**		None	May forget comparable values from prior plan

NOTE: *Definitions for task/knowledge requirements:
[1]*Abstract reasoning*: Process of perceiving issues and reaching conclusions through the use of symbols or generalizations rather than concrete, factual information
[2] *Attentional control:* Controlled processing on difficult tasks or tasks that use unfamiliar items.
[3] *Comparison:* Examination of 2 or more items to establish similarities and differences.
[4] *Declarative knowledge:* Knowledge about facts or things.
[5] *Good visual acuity:* Clarity or sharpness of vision, the ability to see fine detail (e.g., reading test, recognizing symbols).
[6] *Numerical computation:* Ability to solve mathematical equations.
[7] *Reading comprehension:* Ability to understand what is read.
[8] *Visual search:* Ability to actively scan the visual environment for a particular object or feature (target) among other objects or features.
[9] *Working memory:* Brief, immediate memory for material that is currently being processed; a portion of working memory also coordinates ongoing mental activities.
